# Reply to Jobling, “Lysogeny of Escherichia coli by the Obligately Lytic Bacteriophage T1: Not Proven”

**DOI:** 10.1128/mBio.01007-21

**Published:** 2021-05-04

**Authors:** Leanid Laganenka, Victor Sourjik

**Affiliations:** a Institute of Microbiology, D-BIOL, ETH Zürich, Zürich, Switzerland; b Max Planck Institute for Terrestrial Microbiology and LOEWE Center for Synthetic Microbiology (SYNMIKRO), Marburg, Germany; Johns Hopkins Bloomberg School of Public Health

**Keywords:** lysogeny, phage, pseudolysogeny

## REPLY

In his Letter to the Editor (1), Dr. Jobling questions our conclusion that T1 phage is present in a lysogenic state in Escherichia coli ATCC 15144 (2). His primary concern lies in the absence of T1 phage DNA reads in the sequenced total DNA of the E. coli host strain. We believe that such underrepresentation of the phage DNA reads could be explained by the technical aspects of sample preparation and sequencing, since the T1 phage genome is extensively methylated and such methylation can interfere with next-generation sequencing (3, 4). Indeed, next-generation sequencing of low-copy-number episomal phages of Staphylococcus aureus requires prior enrichment of their DNA (5).

As mentioned by Dr. Jobling, the association between the T1 phage and E. coli ATCC 15144 observed in our study was extremely stable, with all colonies remaining positive for phage DNA even after 28-fold single colony purification (see Fig. S2A in reference 2). In our opinion, this all but rules out that the presence of the T1 phage in the culture could be explained by low levels of contamination leading to persistent infection—or else it would require extremely high levels of contamination that would have been easily detected by sequencing; see the point above.

Moreover, the specific nature of the association between the T1 phage and E. coli ATCC 15144 is further consistent with our observation that other E. coli strains do not form similarly stable lysogens, even in cases when formation of unstable lysogens could be observed. Of note, we did confirm that these unstable lysogens are not genetically resistant mutants, since they not only lose the phage over time but also become susceptible to renewed infection. We apologize for not including these data in our publication.

Clearly, further analysis is required to determine the exact nature of the observed lysogenic state of T1 phage in E. coli ATCC 15144, which may also settle the question of its exact classification. In recent decades, the bacteriophage field has been gradually shifting from the classical black-and-white (lytic-lysogenic) definition of bacterium-phage interactions, as it now becomes apparent that various transient nonlytic (pseudolysogenic) and chronic infections are common in nature (6). As mentioned by Dr. Jobling, another recent paper (7) reported a nonlytic phage-carrier state for a T1-like phage in a different E. coli background, suggesting that this state might be pseudolysogenic. In our case, chronic infection or pseudolysogeny in its conventional definition as a coexistence between the phages and the host in a culture (8) could be ruled out by the extremely high stability of the T1 phage interaction with E. coli ATCC 15144 and by the fact that no active virus particles could be detected in uninduced cultures. However, the term “pseudolysogeny” has also been used in a broader sense to describe various metastable phage-host interactions (8), including those where the phage relies on specific molecular mechanisms to ensure stable maintenance of its DNA within the host (9). In this context, the distinction between the episomal lysogeny (10–13) as concluded in our paper and an extremely stable pseudolysogeny might become difficult to see, particularly in the absence of the detailed knowledge of molecular mechanisms involved in the T1 phage maintenance in E. coli ATCC 15144. We hope that the recent surge of interest in understanding the diversity and complexity of bacterium-phage interactions (14) will lead to refinement of the phage classification and enable a less debatable definition for the case of bacterium-phage interactions described in our study.
